# Supplementary material for: Microfluidic-Based Scratch Assays for Wound Healing Studies: A Systematic Review
Source: Cells. 2025 Dec 5;14(24):1931. doi: 10.3390/cells14241931 (PMC12731330; doi:10.3390/cells14241931)
Supplement: Supplementary file 1 [file cells-14-01931-s001.zip › cells-4010448-supplementary.pdf]

SUPPLEMENTARY MATERIALS

Microfluidic-Based Scratch Assays for Wound Healing Studies: A Systematic Review

Fernando A. Oliveira[1,†], Nicole M.E. Valle[1,†], Keithy F. da Silva[1], Arielly H. Alves[1], Marta C. S. Galanciak[1], Gabriel M. Rosário[1], Javier B. Mamani[1], Mariana P. Nucci[2], and Lionel F. Gamarra[1,*]

[1] Hospital Israelita Albert Einstein, 05652-000, São Paulo – SP, Brazil; fernando.ao@einstein.br (F.A.O.); nicolemev@gmail.com (N.M.E.V.); keithyflx@gmail.com (K.F.S.); ariellydahora1997@gmail.com (A.H.A.); marta.caetano.2004@gmail.com (M.C.S.G.); gabrielmrosario16@gmail.com (G.M.R.); javierbm@einstein.br (J.B.M.); lionelgamarra7@gmail.com (L.F.G.)

[2] LIM44 - Hospital das Clínicas da Faculdade Medicina da Universidade de São Paulo, 05403-000 São Paulo – SP, Brazil; mariana.nucci@hc.fm.usp.br (M.P.N.)

* Correspondence: lionelgamarra7@gmail.com; Tel.: +55-11-2151-0243

† These authors contributed equally to this work

Table S1: Complete search strings used in the PubMed, Scopus, and Web of Science databases for conducting the systematic review. Each set of terms was constructed using specific boolean operators to combine keywords related to microfluidics, on-a-chip platforms, and wound-healing assays.

Database	Search strings
**PubMed**	[((((((((microfluidic[Title/Abstract]) OR (microfluidics[Title/Abstract])) OR ("microfluidic device"[Title/Abstract])) OR ("microdevice"[Title/Abstract])) OR ("organ-on-a-chip"[Title/Abstract])) OR ("lab-on-a-chip"[Title/Abstract])) OR ("On-chip"[Title/Abstract])) OR ("wound-on-a-chip"[Title/Abstract])) OR ("microphysiological system"[Title/Abstract])) AND ((((("scratch assay"[Title/Abstract]) OR ("wound-healing"[Title/Abstract])) OR ("wound-healing assay"[Title/Abstract])) OR ("wound healing"[Title/Abstract])) OR ("wound healing assay"[Title/Abstract]))]
**Scopus**	[((TITLE-ABS-KEY (MICROFLUIDIC) OR TITLE-ABS-KEY (MICROFLUIDICS) OR TITLE-ABS-KEY ("MICROFLUIDIC DEVICE") OR TITLE-ABS-KEY (MICRODEVICE) OR TITLE-ABS-KEY ("ORGAN-ON-A-CHIP") OR TITLE-ABS-KEY ("LAB-ON-A-CHIP") OR TITLE-ABS-KEY ("ON-CHIP") OR TITLE-ABS-KEY ("WOUND-ON-A-CHIP") OR TITLE-ABS-KEY ("MICROPHYSIOLOGICAL SYSTEM"))) AND ((TITLE-ABS-KEY ("SCRATCH ASSAY") OR TITLE-ABS-KEY ("WOUND-HEALING") OR TITLE-ABS-KEY ("WOUND-HEALING ASSAY") OR TITLE-ABS-KEY ("WOUND HEALING") OR TITLE-ABS-KEY ("WOUND HEALING ASSAY")))]
**Web of Science**	[AB=(microfluidic OR microfluidics OR "microfluidic device" OR microdevice OR "organ-on-a-chip" OR "lab-on-a-chip" OR "On-chip" OR "wound-on-a-chip" OR "microphysiological system") AND AB=("scratch assay" OR "wound-healing" OR "wound-healing assay" OR "wound healing" OR "wound healing assay")]

Table S2: Proposal, evaluation, advantages, disadvantages and outcome of wound healing scratch assays in microfluidic devices.

Study	Objective	Evaluation technique	Software	Evaluation time (h)	Advantages	Disadvantages	Outcome
**Scratch assay: Enzymatic cell depletion**							
Moghadam et al. [46]	Develop and assess a microfluidic device to measure BV2 cell adhesion and migration under chemical and mechanical stimuli.	Optical microscopy	ImageJ	0, 6, 12, 18 and 24	Uses gravity for flow. Compares chemical vs. mechanical stimuli in migration. Low-cost, flexible design.	Intense mechanical forces as in the PBS assay can impair cell migration. Excessive width of the lateral channel reduces cell migration.	Chemical wounds promote greater cell migration than mechanical wounds or scratch wounds. Wider channels reduce migration, showing geometry affects cell behavior.
Moghadam et al. [45]	Develop a gravity-driven device to study BV2 cell migration on varied substrates, simulating near in vivo conditions.	Optical microscopy	ImageJ	0, 12, 24 and 48	Creates wounds without ECM loss, unlike traditional assays. Gravity-driven, no pumps. Preserves coating, mimicking in vivo conditions.	Without direct comparison to actual inflammatory conditions of the CNS, it remains a simplified in vitro model.	It was observed that PLL and gelatin stimulated migration, while substrates with lower elasticity, such as PDMS, also promoted greater migration compared to glass.
Zhang et al. [49]	Evaluate how hypoxic MSC medium and biomechanical stimuli reprogram endothelial metabolism to promote angiogenesis.	Fluorescence microscopy (Ki67)	Fiji; Ibidi Chemotaxis and Migration Tool	0 and 6 (time-lapse)	Allows simulation of an in vivo microenvironment by combining shear stress and hypoxia, essential for mimicking vascular repair.	The complexity of the in vivo environment with inflammation and multiple cell types is still not fully modeled.	Conditioned medium plus shear stress boosted endothelial migration, proliferation, and metabolism, promoting angiogenesis for bone repair.
Yang et al. [44]	To develop a passive microfluidic platform to study endothelial wound healing in response to shear stress, overcoming limitations of existing devices.	Optical microscopy	ImageJ	0 and 12	Uses a passive siphon pump, no active pumps needed. Simple, low-cost design with stable, continuous flow and easy operation.	Long-term use limited (up 12 h with 300 mL of medium); flow control via siphon requires precise calibration and has not yet been tested in 3D/OoC	Cell wound healing is accelerated under shear stress, demonstrating the platform's potential for quantitative studies of endothelial wound healing.

Gupta et al. [27]	To develop a microfluidic device to study wound healing in fibroblasts under the influence of shear stress.	Optical microscopy	ImageJ	0, 12 and 24	Simulates a 3D environment like the ECM. Allows precise control of shear forces.	High shear stress risks cell detachment. Sensitive to flow conditions. Limited medium volume restricts long-term experiments.	The optimal shear stress (10 μL/min) accelerated wound healing, while higher rates (20 μL/min) impaired cell migration.
Shih et al. [37]	Study effects of oxygen levels and drugs (Cytochalasin-D, YC-1) on HUVEC migration and proliferation via microfluidics.	Optical and fluorescence microscopy (FD-FLIM)	ImageJ	0, 8 and 16 (time-lapse)	Laminar flow patterns standardize cell areas, work in regular incubators, and enable stable oxygen gradients by confined reactions.	Passive environmental control, limitations in the extent, duration of gradients, and possible variability in response along the channel.	Cell migration toward regions of lower O_2 tension, independent of drug treatments (cytochalasin-D or YC-1).
Lin et al. [31]	Investigate cell migration in response to chemical (β-lapachone) and physical (shear stress and wound width) stimuli in the microfluidic device.	Optical microscopy	Image J	0, 3, 6, 12 and 24	Creates multiple wounds (3-6 mm) in a closed, dynamic setup mimicking in vivo conditions without mechanical cell damage.	Only 3 wound widths available. Can't vary width and shear stress independently. Cell signaling between wounds may affect results.	Healing rose with shear stress and width, but at 0.172 mPa rate was width-independent. β-lapachone (≤0.5 μM) showed no effect.
Lee et al. [30]	Study the effects of ECM topography on wound healing using an in vitro microfluidic model with nanopatterned surfaces.	Optical and fluorescence microscopy (F-actina/DAPI)	Image J	0, 2, 4, 6, 8, 10, 12, 14, 16, 18 and 20	Ability to precisely control surface topography using nanopatterns. Low cost and high reproducibility for large-scale studies.	Requires strict control of experimental conditions, such as fluid flow and cell culture conditions.	The 1:2 nanostructures sped up migration; 90° alignment boosted healing. Surface topography affected cell orientation and elongation.
Wei et al. [42]	Develop a tubing-free assay to measure smooth muscle cell migration under varied geometry, coatings, and chemokines.	Optical microscopy	ImageJ	0, 3, 6, 12 and 24	Eliminates the need for external pumps. Low sample consumption. Standardized wound generation.	Need for adaptation for high-throughput assays.	Migration increased with taller channels, collagen coating, and PDGF-BB, especially in primary vascular smooth muscle cells.
Xi et al. [43]	Develop a microfluidic method to create wounds with defined edges and study cell migration in response to biochemical factors.	Optical microscopy	Image-Pro Plus	0, 2, 4, 6, 13, and 20	Precise wounds without cellular damage.	Dependence on controlled laminar flow.	FGF-2.IGF-I.PDGF-BB.and vitamin C promoted migration. While dexamethasone inhibited.

Felder et al. [8]	Evaluate the effect of HGF on alveolar epithelial regeneration.	Optical, fluorescence microscopy (DAPI and occludin) and TEM	ImageJ	0, 2, 5, 9, and 13 (time-lapse)	Creates controlled wounds via focused flow, with less damage than scratches. Works with few cells, ideal for personalized medicine.	Complexity in the fabrication and operation of the microfluidic device. Specific equipment and technical expertise required.	HGF boosted healing via proliferation, spreading, and migration. More reproducible than scratch assay; promising for personalized use.
Murrell et al. [32]	Develop a microfluidic method to isolate effects of space and damage on epithelial migration, assessing physical and chemical cues.	Optical and fluorescence microscopy (Live-dead)	ImageJ, Imaris and Matlab	36 (time-lapse)	Precise wound without cell damage (using trypsin). Spatial and temporal control of chemical gradients.	Complexity of the microfluidic device. Limitation in the analysis of submarginal cells.	Free space is sufficient to induce migration at the edge, but the motility of submarginal cells is reduced; ROS inhibits migration.
Huang et al. [28]	Analyze the effect of ethanol on the migration of human breast cancer MCF-7 cells using a microfluidics-based wound healing assay.	Optical microscopy	Image-Pro Plus	0, 2, 4, 8, 16 and 24	Generates precise wounds with intact borders via laminar flow. Biomimetic control reduces variability.	Need for specialized equipment (microdevice, flow system). Complexity in preparation and operation of the microfluidic system.	Ethanol increased cell migration in a concentration-dependent manner.
van der Meer et al. [41]	Develop a microfluidic wound-healing assay to quantify endothelial cell migration and compare it with the conventional assay.	Optical and fluorescence microscopy (Live-dead and phalloidin)	ImageJ	0, 1, 2 and 3	Generation of stable growth factor gradients. Controlled application of shear stress. Requires small amounts of cells and reagents.	Migration rate lower compared to the conventional assay (2x slower). Requires expertise in microfluidics and specialized equipment.	The VEGF165 gradient doubled cell migration, while shear stress increased it by 1.8 times.
Nie et al. [34]	Create an on-chip migration assay to cut cell/reagent use and allow scaling for high-throughput screening.	Optical microscopy	NR	24	Laminar trypsin flow precisely controls wound edges, spares surrounding cells, and cuts reagent and cell use.	Microfluidic device fabrication needs precision and specialized gear, challenging labs without microfluidics expertise.	It demonstrated that microfluidic assay is effective for studying cell migration and testing the effects of promoters/inhibitors.
**Scratch assay: Physical cell depletion**							
Chen et al. [9]	Evaluate the shear stress generated by flow in various directions on wound closure	Optical microscopy	Cellpose and ImageJ	0 and 12	Low-cost, pump-free device mimics in vivo better than 2D assays with unidirectional flow; enables	Efficacy depends on the angle between flow and wound. Less precise flow	Wound healing increased by 76% ± 6% with flow at 45° relative to the wound axis.

	speed using a pump-free microfluidic device.				flow effect studies on healing.	control compared to pump-based systems.	
Moghadam et al. [46]	Develop and assess a microfluidic device to measure BV2 cell adhesion and migration under chemical and mechanical stimuli.	Optical microscopy	ImageJ	0, 6, 12, 18 and 24	Uses gravity for flow. Compares chemical vs. mechanical stimuli in migration. Low-cost, flexible design.	Intense mechanical forces as in the PBS assay can impair cell migration. Excessive width of the lateral channel reduces cell migration.	Chemical wounds promote greater cell migration than mechanical wounds or scratch wounds. Wider channels reduce migration, showing geometry affects cell behavior.
Shaner et al. [36]	Investigate the effects of direct current electrical stimulation on wound healing in healthy and diabetic human keratinocyte models.	Optical microscopy	ImageJ (kymograph Builder) and CellTracker	12 (time-lapse)	Controls electric field precisely; non-metallic electrodes avoid corrosion, work without salt bridges. Useful for disease models and co-cultures.	Complexity in current standardization. Requires specialized equipment for device fabrication.	A unidirectional electric field accelerates wound closure up to 3 times, showing that besides electrotaxis, pathways like EGFR and MAPK are also involved.
Yin et al. [44]	Develop a biomimetic, automated, and highly reproducible microfluidic device for studying cell migration under various shear conditions.	Optical and fluorescence microscopy (Live/Dead)	ImageJ ou Fiji (Wound Healing Analysis)	24 (time-lapse)	Simulates physiological mechanical stress with high uniformity. Handles ~400 samples simultaneously with real-time cell migration monitoring.	Doesn’t monitor pH, crucial for drug effects. Uses image sensors. Tested mainly on cancer cells; other cell types not yet evaluated.	The device demonstrated effectiveness in combined chemotherapy studies (CisPt + DOX) and antiangiogenesis, showing distinct cellular responses under shear stress.
Yilmaz et al. [47]	Create wounds in microfluidic cell cultures via microrobotics for standardized migration analysis without harming cell viability.	Optical microscopy	Image J	0, 4, 8, 12, 16 and 20 or 0, 6, 12, 18, 24 and 30	Creates uniform wounds of varied shapes without chemicals; compatible with many microfluidic systems.	Needs microrobot microfabrication. Tested only in 2D cultures. Relies on external control; not fully automated yet.	Microrobot speed migration vs. micropipette. Wound shape affected healing: cross-shaped closed in 20h, triangles took 30h.
Gupta et al. [27]	To develop a microfluidic device to study wound healing in fibroblasts under the influence of shear stress.	Optical microscopy	ImageJ	0, 12 and 24	Simulates a 3D environment similar to the ECM. Allows precise control of shear forces.	High shear stress risks cell detachment. Sensitive to flow conditions. Limited medium volume restricts long-term experiments.	The optimal shear stress (10 μL/min) accelerated wound healing, while higher rates (20 μL/min) impaired cell migration.
Monfared et al. [35]	Develop a microfluidic device for automated, miniaturized,	Optical and fluorescence	Image J	0, 4, 8 12, 16 and 20	High reproducibility and precision in creating	Xurography has lower resolution when compared	The device proved effective in evaluating the effects of bFGF (an

	reproducible study of human dermal fibroblast migration under bioactive substances.	microscopy (Live-dead and phalloidin)			circular wounds (compared to conventional methods).	to photolithography or CNC techniques.	11.87% increase in wound closure) and MMC (a 36.57% inhibition).
Go et al. [26]	To study the wound healing processes of fibroblastic cells after wound formation in a microfluidic device.	Optical microscopy	Image Pro-Plus	192 (12 h interval)	Simple and easy-to-use device. Capable of applying external pressure to create wounds and control wound area formation.	Only applicable for 2D culture. Lower healing efficiency for larger wounds.	Smaller (400 μm) wounds healed faster with non-linear cell growth. Method is reproducible and effective for studying migration and proliferation.
Sticker et al. [38]	Develop a microfluidic device to create circular mechanical wounds with high reproducibility.	Optical and fluorescence microscopy (GFP and EthD-1)	ImageJ and Adobe Photoshop	0, 6, 12 and 24	Highly reproducible circular wounds. Preservation of the ECM. Removal of cellular debris by laminar flow.	The system requires specialized pneumatic equipment and involves complex device fabrication.	Cell migration was 2.5x faster than scratch assay. TNF-α cut healing by 42%; Mitomycin-C blocked proliferation and migration.
Uhlig et al. [40]	Develop a thermoresponsive coating to precisely control cell adhesion/detachment in scratch assays with high reproducibility and resolution.	Optical and fluorescence microscopy (Calcein)	Cell-R	0, 5, 10 and 15	Gentle and reversible cell detachment through thermal control. Prevents cellular damage common in invasive methods.	Needs temperature control (specific setup). Not suitable for all cells or substrates without modifications.	Thermoresponsive microgels enable gentle, localized cell detachment with high viability and clear wound edges for precise assays.
Handly et al. [33]	Investigate the limits of paracrine communication in the fidelity of cellular response during wound healing.	Optical and fluorescence microscopy (Ca2+ and ERK)	MATLAB	0.5 (time-lapse)	Generates controlled wounds in epithelial layers with reproducible spatial analysis of Ca^{2+} and ERK via real-time fluorescence microscopy.	In vitro results may not reflect complex in vivo tissues. Needs specialized microfluidics, fluorescence microscopy, and uses fluorescent sensors with limits.	EGF via P2Y receptors and ATP boosts cell response by cutting noise, effective within ~100 μm, showing paracrine signaling range's importance.
An et al. [24]	Study interactions cells with microenvironment and each other during wound healing using micropillar substrates.	Optical and fluorescence microscopy (CellTracker)	Image-Pro Plus and SPSS	0, 4, 12, 24 and 36 (time-lapse)	Microfluidic device with micropillar substrate enables coculture studies and controlled wounding.	Device fabrication complexity and requirement for specialized equipment.	Fibroblasts contributed more to wound healing in coculture. Micropillar topography affected cell morphology and migration.
Sun et al. [39]	Develop and utilize an electrically stimulated	Optical microscopy	ImageJ	2 (time-lapse)	Cost-effective and high-throughput device. Mimics	Does not fully replicate all complexities of the in vivo	The device demonstrated that both the electric field and β-lapachone

	microfluidic wound healing chip.				the in vivo microenvironment and enables electric field treatment of wounds.	physiological environment. Requires temperature and flow control.	promote wound healing. However, excessive doses can be harmful due to ROS production.
**Scratch assay: Physical cell exclusion**							
Yin et al. [44]	Develop a biomimetic, automated, and highly reproducible microfluidic device for studying cell migration under various shear conditions.	Optical and fluorescence microscopy (Live/Dead)	ImageJ or Fiji (Wound Healing Analysis)	24 (time-lapse)	Simulates physiological mechanical stress with high uniformity. Handles ~400 samples simultaneously with real-time cell migration monitoring.	Doesn't monitor pH, crucial for drug effects. Uses image sensors. Tested mainly on cancer cells; other cell types not yet evaluated.	The device demonstrated effectiveness in combined chemotherapy studies (cisplatin + doxorubicin) and antiangiogenesis, showing distinct cellular responses under shear stress.
Imashiro et al. [29]	Investigate how propagated surface acoustic waves affect directional fibroblast migration, considering variations in wave intensity.	Optical and fluorescence microscopy (DAPI and Actina)	Fiji	0, 2, 4, 6 and 8 (time-lapse)	Wave direction aligned with migration allows precise analysis. Non-conductive substrate avoids interference; temperature control prevents damage.	High wave intensity may cause delamination and inhibit migration. Vertical forces and elevations affect adhesions. Device is complex.	Acoustic waves at critical intensity enhance cell migration, while higher intensities suppress migration.
Sticker et al. [38]	Develop a microfluidic device to create circular mechanical wounds with high reproducibility.	Not reached	Not reached	Not reached	Not reached	Not reached	Not reached
Gao et al. [25]	To evaluate the effects of dual BRAFV600E (vemurafenib) and EGFR (gefitinib) inhibition on melanoma cell migration via microfluidics.	Optical and fluorescence microscopy (phalloidin)	ImageJ	0, 12, 24 and 48	Low cost. Minimal sample consumption (20 μL). Standardized wound generation without chemicals or mechanical force.	Requires manual pressure adjustment for flow control.	Vemurafenib and gefitinib synergistically inhibited migration in MV3 cells, showing the device's use for rapid, personalized testing.
Zhang et al. [48]	Evaluate migration and proliferation in a microfluidic wound model, focusing on EGF effects and MSCs migration toward tumor cells.	Optical and fluorescence microscopy (CellTracker, Ki67/DAPI)	NR	64 (8 h interval)	High reproducibility. Creation of multiple cell-free regions with uniform size and shape. Simple and low-cost process.	Two-dimensional model that may not fully replicate the physiological 3D environment.	EGF boosts GES-1 migration; MSCs migrate more with tumor cells than GES-1. Validates a high-throughput platform for migration studies.

Abbreviations - PBS: Phosphate-Buffered Saline; ECM: Extracellular Matrix; CNS: Central Nervous System; PLL: Poly-L-Lysine; PDMS: Polydimethylsiloxane; MSCs: Mesenchymal Stem Cell; 3D: Three-Dimensional; QoC: Quality of Care; HUVEC: Human Umbilical Vein Endothelial Cells; FD-FLIM: Fluorescence Lifetime Imaging Microscopy (Frequency-Domain); DAPI: 4′,6-Diamidino-2-Phenylindole; PDGF-BB: Platelet-Derived Growth Factor-BB; FGF-2: Fibroblast Growth Factor-2; IGF-I: Insulin-Like Growth Factor-I; HGF: Hepatocyte Growth Factor; TEM: Transmission Electron Microscopy; ROS: Reactive Oxygen Species; MCF-7: Michigan Cancer Foundation-7 (Human Breast Cancer Cell Line); VEGF165: Vascular Endothelial Growth Factor-165; NR: Not reported; 2D: Two-Dimensional; EGF: Epidermal Growth Factor; EGFR: Epidermal Growth Factor Receptor; MAPK: Mitogen-Activated Protein Kinase; DOX: Doxorubicin; CisPt: Cisplatin; bFGF: Basic Fibroblast Growth Factor; MMC: Mitomycin C; GFP: Green Fluorescent Protein; EthD-1: Ethidium Homodimer-1; TNF-α: Tumor Necrosis Factor-alpha; Ca2+: Calcium Ion; ERK: Extracellular Signal-Regulated Kinase; P2Y: Purinergic Receptor P2Y; ATP: Adenosine Triphosphate; SPSS: Statistical Package for the Social Sciences; GES-1: Gastric Epithelial Cell Line-1.
